# Experimental Theory of the Formation of Bone

**Published:** 1847-10

**Authors:** 


### ART. IX.

1. *Théorie Expérimentale de la Formation des Os.* Par P. FLOURENS, Secrétaire perpétuel de l'Académie Royale des Sciences, Professeur de Physiologie comparée au Muséum d'Histoire Naturelle de Paris, &c. &c. Avec 7 Planches gravées.—*Paris,* 1847.

*Experimental Theory of the Formation of Bone.* By P. FLOURENS, Perpetual Secretary of the Royal Academy of Sciences, Professor of Comparative Physiology in the Museum of Natural History of Paris, &c. &c. With Seven Engravings.—*Paris,* 1847. 8vo, pp. 164.

2. *Elements of Anatomy.* By JONES QUAIN, M.D. Fifth Edition. Edited by RICHARD QUAIN and WILLIAM SHARPEY, M.D., Professors of Anatomy and Physiology, University College, London. Illustrated by numerous Engravings on Wood. Parts I and II.—*London,* 1843-6. 8vo, pp. 1063.

3. *Cyclopædia of Anatomy and Physiology.* Edited by ROBERT B. TODD, M.D., F.R.S., Professor of Physiology and of General and Morbid Anatomy in King's College, London, &c. &c. Article, *Osseous Tissue;* by JOHN TOMES, Surgeon-Dentist to the Middlesex Hospital.—*London,* 1846. Illustrated with numerous Engravings.

THE history of the formation, growth, and regeneration of bone has long been favourite subjects of inquiry, not only among anatomists and physiologists, but also among such practical surgeons as usually care little for such investigations. There are many reasons why this should be the case, sufficiently obvious to prevent the necessity of our dwelling on them. A vast number of observations and experimental results have been laid before the world at different times, by men of very different qualifications and habits of mind; and there has been accordingly much discrepancy of opinion on many of the questions which the inquiry involves, and no inconsiderable amount of dogmatism has been manifested in support of the various positions advanced. In this instance, as in many others, however, the progress of knowledge and the prevalence of more philosophical habits of thought have tended to reconcile views which formerly seemed altogether opposed to each other ; showing that the facts on which they were respectively based were in reality perfectly consistent, and that it was only in the exclusiveness of the deductions from them that those deductions were erroneous.

Of the attention which has recently been bestowed upon microscopic research, the osseous texture has come in for its full share. Here, too, many errors of observation were at first committed, which have since undergone correction; and many opinions were founded upon them, which have since been modified or abandoned. There has been obviously increasing tendency to agreement on many points which were at first matters of dispute; whilst new questions have been opened up in the more recondite parts of the inquiry; and various opinions which were the result of too limited an induction have been found to be no longer tenable, although the authority with which they were at first advanced, has caused them to be very generally admitted.

We propose, on the present occasion, to put our readers in possession of the results of those later inquiries on this subject, which have most tended to modify the views originally advanced regarding the structure and development of bone; and we shall then examine the results of the experimental inquiries of M. Flourens, with the view of contrasting their results with those at which we arrive by a much simpler method of inquiry, and of pointing out what we believe to be a gross exaggeration of his claims as an original discoverer in this department of physiology.

The elementary substance of bone, considered in its simplest aspect, appears to be homogeneous, even when examined with high powers of the microscope. Mr. Tomes, however, has directed attention to the fact that it possesses a minute granular structure.

"For the purpose of examination, it is best to take a very small portion of a thin plate of bone; such may be found in the ethmoid bone of small animals, as of the rat. If the piece be well selected, it will be found to contain no Haversian canals or corpuscles, but to be extremely thin and transparent. Such a portion, when viewed with the 1-8th of an inch object glass, will present a delicate granular aspect with the surface nodulated. This granular appearance arises from the substance of the bone being composed of minute irregularly spherical granules. It is not difficult to trace this structure in any specimen of bone, though in some it is much more distinct than in others...... ... The granules may be obtained separated from each other, so that each individual may be examined apart from its fellows. When so exposed to view, they exhibit a tolerably regular character, being mostly spherical, some few having an oval form. In some specimens, the oval predominates over the spherical conformation. Often a few will be found which are egg-shaped, with the smaller end elongated, though to no great extent. The osseous granules may be gained by subjecting bone to high-pressure steam, or to a red heat, till all the animal matter is removed. In either instance, the granules may be obtained by taking a small portion of the so-treated bone, saturating it with water, and then gently reducing it to a powder between the slips of glass. By this manipulation the granules individually will be rendered evident when the specimen is examined under a high power............The granules themselves are subject to some variety in size, commonly varying from one sixth to one third the size of a human blood-globule." (Cyclop. of Anat. and Phys., vol. iii, p. 848.)

The following observations by Mr. Tomes, on the ossification of permanent cartilage, are of much interest in connexion with the foregoing statements. He has particularly observed the process as it occurs in the cartilages of the larynx, where it takes place slowly, and with no preliminary change in the cartilage. Osseous granules are developed in the intercellular substance, only a few of them being at first seen, and these being spherical and isolated; but a much larger number gradually present themselves, which unite to form an osseous mass.

"The formation of the individual granules is more readily observed in these cartilages than in any other situation. This form of ossification establishes an interesting and explanatory link of connexion between bone and the various osseous plates we find in abnormal situations. For in the latter the spherical granules appear; and these, at first few and isolated, and lying among the fibres of the tissue, rapidly increase in number, unite and form an osseous mass. Osseous plates occur in various soft tissues as the result of deranged action, where in the healthy condition of the part they are not found. Thus we have osseous plates formed in the coats of arteries, in the pleura, in the diaphragm; also osseous masses in the uterus, and sometimes in the muscular tissue and in the placenta. These plates are all formed in the same manner, namely by the development of minute spherical osseous granules, which form into a mass, the shape of which is modified by the form of the tissue in which the development occurs." (Cyclop. of Anat. and Physiol., vol. iii, p. 857.)

This granular arrangement, however, appears to be characteristic rather of the mineral than of the organic constituent of bone; for it cannot be traced in the gelatinous basis, which is left after the earthy matter has been removed by the agency of an acid; on the contrary, this appears from the observations of Dr. Sharpey, which we have ourselves confirmed, to have a *fibrous* structure. If a piece of a long bone be steeped in dilute acid until its calcareous matter is removed, thin films may be peeled off from it with a little care in a longitudinal direction. Most of these fibres however, consist of several superimposed laminæ; and it is only by looking towards the edge, where the different layers are usually torn unequally, so that some extend beyond others, that we can find parts thin enough to show the real structure.

"It will then appear plainly that they are made up of transparent fibres, decussating each other in the form of an exceedingly minute network. The fibres intersect obliquely, and they seem to coalesce at the points of intersection, for they cannot be traced out from one another; but at the torn edge of the lamella they may often be seen separate for a little way, standing out like the threads of a fringe. Most generally they are straight; but they are not always so, for in some parts they assume a curvilinear direction. Acetic acid causes these fibres to swell up and become indistinct, like the white fibres of cellular (areolar) and fibrous tissue." (Quain's Anatomy, p. 143.)

Hence it would seem inappropriate to designate the organic basis of bone by the term cartilage; for it has nothing of the structure of true cartilage; and it differs also in chemical composition, being composed like the ordinary fibrous tissues of gelatin, whilst cartilage, whether temporary or permanent, is made up of chondrin. The prevalent notion of ossification has been, that the calcareous salts are deposited in the substance of the previously existing cartilage; so that the latter, consolidated by the ossifying process, continues to exist in bone. We shall presently see, however, that there is much reason to believe the osseous tissue to be entirely a new formation; the previous cartilage (where it exists) being merely a temporary substitute for bone, serving at the same time as a sort of mould for giving shape to the bony deposit, and as a support to the still softer parts of the fabric.

In the midst of the dense substance of all but the thinnest laminæ of bone, we find certain dark spots with thread-like lines radiating from them: these, when first discovered, were named the osseous corpuscles, and were supposed to be solid collections of calcareous matter, to the opacity of which their dark appearance, under transmitted light, was supposed to be due. We believe, however, that all microscopists (in this country at least) are now agreed that these spots are *excavations* in the solid osseous substance, and that the thread-like lines are minute tubes or *canaliculi* proceeding from them. This is most conclusively proved by the passage of fluids through them, which may be distinctly seen to take place under the microscope when a dry section of bone is moistened with oil of turpentine. These *lacunæ* (to use the term first introduced by Messrs. Todd and Bowman) seem, in the recent bone, to contain a soft granular matter, which may possibly be a persistent cell-nucleus, and may have an important function in selecting the material which the canaliculi are destined to convey. The manner in which the canaliculi present themselves to the eye, when they are looked for in thin lamellæ torn from the decalcified animal basis, is thus described by Dr. Sharpey:

"The lamellæ are seen to be perforated with fine apertures placed at very short distances apart. These apertures were described by Deutsch, but they have not much attracted the notice of succeeding observers; they appear to me to be the transverse sections of the canaliculi already described, and their relative distance and position accord sufficiently with this explanation. According to this view, therefore, the canaliculi might (in a certain sense) be conceived to result from the apposition of a series of perforated plates, the apertures of each plate corresponding to those of the plates contiguous with it; in short, they might be compared to holes bored to some depth in a straight or crooked direction through the leaves of a book, in which case it is plain that the perforations of the adjoining leaves would correspond." (Quain's Anatomy, p. 144.)

When the lamella is thin enough for its fibrous structure to be distinguished, it may be seen that the perforations correspond to the intervals or openings between the reticulated fibres; and on this view the canaliculi are to be regarded as little vacuities left in the tissue during the deposition of the fibres, resembling the open figures left out in the weaving of some artificial fabrics.

Much attention has lately been given, especially by Mr. John Quekett, to the form and dimensions of the lacunæ, and to the mode in which the canaliculi radiate from them; as indicative of the class and order of vertebrated animals to which any particular bone may belong. It must be obvious that, if any distinction of this kind can be established, it will be most valuable in aiding in the determination of fossil remains, whose fragmentary condition prevents their character from being recognised by their external configuration; whilst it will afford an additional means of deciding upon the zoological position of a doubtful animal, such as the lepidosiren, whose structure is intermediate between that of two classes of vertebrata. But we cannot attach any great importance to this test, until a much more complete examination shall have been made of the variations which may present themselves in the form, size, and arrangement of the lacunæ in any one animal; since it is quite possible that different portions of the same skeleton may present differences in these characters which we should at first be inclined to regard as diagnostic of difference of class.

We shall now direct the attention of our readers to the recent progress of knowledge in regard to the process of ossification, which has been the fruit of carefully conducted microscopic research. It was very natural that in studying this process, attention should be at first almost exclusively fixed upon that phase of it which presents itself in the consolidation of the long bones of young animals, which are at first laid down or moulded (so to speak) in cartilage; and that hence the *intra-cartilaginous* mode of ossification should be for a time the only one minutely described. Even this, however, is not yet fully understood; and for the due comprehension of it, we deem it requisite that the *intra-membranous* form of ossification, to which attention has lately been recalled by Dr. Sharpey, should be first elucidated. We shall therefore bring his observations under the notice of our readers in the first instance; and may state *in limine* that we have ourselves repeated and confirmed them in almost every particular.

That osseous tissue may be developed in the substance of fibrous membranes, is a fact well known to every pathologist; but the occurrence of such a method of development, as a part of the regular process of osteogeny in man and other vertebrata, has been commonly lost sight of; although it was clearly distinguished from the intra-cartilaginous method of ossification, more than a hundred years since, by Dr. Nesbitt, and has been since alluded to more or less distinctly by other writers on the subject. Dr. Sharpey is the first microscopist, however, so far as we are aware, who has applied himself to the determination of the minute stages of this process; which may be best studied in the tabular bones of the fœtal cranium. The base of the skull in the human embryo is cartilaginous; but in the roof we find (except where there happen to be commencing muscular fibres) only the integuments, the dura mater, and an intermediate layer of fibrous membrane in which the ossification proceeds. In the embryo of the sheep, the roof of the cranium is strengthened by cartilaginous plates which rise up from the base of the skull; but these are evidently destined only to afford a temporary support, and are not in any way subservient to the ossifying process, which takes place in the membrane on its exterior.

"The commencing ossification of the parietal bone, which may be selected as an example, appears to the naked eye in the form of a network, the little bars or spicula of bone running in various directions, and meeting each other at short distances. By and by, the ossified part becoming extended, gets thicker and closer in texture, especially towards the centre, and the larger bony spicula which now appear, run out in radiating lines to the circumference; the ossification continuing thus to spread and consolidate until the parietal meets the neighbouring bones, with which it is at length united by suture.

"When further examined with a higher magnifying power, the tissue or membrane in which the ossification is proceeding appears to be made up of fibres and granular corpuscles, with a soft, amorphous, or faintly granular uniting matter.

The fibres have the characters of the white fibres or rather fasciculi of the cellular (areolar) and the fibrous tissue, and are similarly affected by acetic acid. The corpuscles are, for the most part, true cells, with an envelope and granular contents; some about the size of blood-particles; but many of them two or three times larger. In certain parts the fibres, but in most the corpuscles, predominate; and on the whole the structure might be said to be not unlike that of a fibrous membrane in an early stage of development. The bone, seen by transmitted light, is dark and opaque; and near the growing edge it is decidedly granular.

"On now observing more closely the bony processes or spicula at the circumference, where they shoot into the membrane, it will be seen, as you trace them into the soft tissue, that they gradually lose their opaque and granular character, indicative of earthy impregnation, and are prolonged a little way into the membrane, in form of bundles of transparent fibres, having all the characters of those of fibrous tissue. Those fibres are in some parts closely gathered into thick bundles; but more generally the fasciculi are smaller, and irregularly interlaced or reticulated, with corpuscles lying between them; and we may often observe that where the earthy deposit is advancing to invade the fibres, the recently and as yet imperfectly calcified bone with which they are continuous, presents a similar open and coarsely reticular structure; though the older, harder, and more opaque part is comparatively solid and compact. The appearance referred to is especially well seen at those places where a cross bridge of bone is being formed between two long spicula; we may there distinguish the clear soft fibres which have already stretched across the interval; and the dark granular opacity indicating the earthy deposit may be perceived advancing into them, and shading off gradually into their pellucid substance, without a precise limit.

"It thus appears that in the intra-membranous ossification, the growing bone shoots into the soft tissue, in form of transparent fibres, resembling those of fibrous texture, more or less intermixed with granular corpuscles; and that these fibres become charged with earthy salts. As to the cells or corpuscles, they certainly seem in some way involved in the ossification along with the fibres; but I am not able to say what precise share they have in the process. It has been supposed that they eventually form the lacunæ of the bone; but we shall enter upon this question afterwards.

"As the bone extends in circumference, it also increases in thickness; the vacuities between the bony spicula become narrowed or disappear; and at a more advanced period the tabular bones of the cranium are tolerably compact towards the centre, although their edges are still formed of slender radiating processes. At this time, also, numerous furrows are grooved on the surface of the bone in a similar radiating manner; and towards the centre these are continued into canals in the older and denser part, which run in the same direction. The canals, as well as the grooves, which become converted into canals, contain blood-vessels supported by processes of the investing membrane, which deposit concentric layers of bone within; and when thus surrounded with concentric laminæ, these tubular cavities are in fact the Haversian canals." (Dr. Quain's Anatomy, p. 151.)

This account of the first formation of osseous tissue harmonizes extremely well with the description already given of the fibrous texture of the animal basis left after the removal of the calcareous salts by acid; and it would seem, therefore, correct to say, of *this* form of osseous substance at any rate, that it is a fibrous tissue impregnated with calcareous salts, which have entered into chemical combination with the gelatine of which the fibres are composed. Dr. Carpenter has pointed out (Human Physiology, § 192,) the remarkable confirmation which this view derives from an examination of the skeletons of the Echinodermata; these being unquestionably composed of a *calcified areolar tissue,* which presents a striking resemblance to the first osseous network visible in the intramembranous process of ossification, and which differs from bone chiefly in having its development arrested at that point, so that it remains incapable of modification, and never passes on to any higher form of organization.

With respect to the early stages of the intra-cartilaginous form of ossification, we believe that there is no considerable difference of opinion amongst microscopists. A *vertical* section of the cartilage carried down to the plane of ossification, shows the cartilage-cells arranged in rows or oblong groups, between which the transparent matrix or intercellular substance appears in the form of clear longitudinal lines. The peculiar arrangement of the cartilage-cells is probably due to their rapid multiplication in the neighbourhood of the seat of ossification, which is copiously supplied with blood-vessels; each oblong group consisting of a row of cells, which seems to have been formed by the usual process of cell-growth, from a single individual. When a *transverse* section of the ossifying cartilage is made, parallel to the plane of ossification and at a short distance from it, we find that the matrix forms a network with large circular areolæ, which are occupied by the cartilage-cells, here in much nearer approximation than in ordinary cartilage. Now it is in the matrix or intercellular substance of the cartilage, that the first calcareous deposit takes place; and it is seen in a vertical section shooting upwards in thin cylindrical plates in the clear spaces of the cartilage, between the oblong groups of cells whose lower ends it envelopes; whilst in a transverse section, carried partly through the bone and partly through the cartilage, we can as distinctly trace the transition from the calcified to the simply chondrinous network of intercellular substance, as we can in intra-membranous ossification from the calcified to the purely gelatinous fibre. The first osseous tissue formed in cartilage, therefore, has, like that formed in membrane, an open areolar texture; but the areolæ at the junction of the bone and cartilage are deep pits or alveoli, into which the ends of the groups of cartilage-cells are received. At a short distance below the ossifying surface, however, where the process has advanced further towards the formation of the permanent bone, the state of things is very different. The first detailed account of the changes which the microscope enables us to trace, as we pass from the ossifying surface towards the perfect bone, was given in the valuable 'Physiological Anatomy' of Messrs. Todd and Bowman, whose statements were chiefly founded on the authority of Mr. Tomes; and the same account is repeated, with little variation in the article "Osseous Tissue," contributed by the latter gentleman to the 'Cyclopædia of Anatomy and Physiology.' Some important modifications have been introduced, however, by Dr. Sharpey; and as we here, also, find our own observations in accordance with his on almost every point, we shall regard his statements as conveying our own conclusions, except where the contrary is expressed.

"According to my own observations, the primary areolæ of bone open into one another by absorption of their intermediate walls, both laterally and longitudinally; they possibly expand to a certain extent, but it is mainly to their lateral confluence that the formation of the larger or what might be termed secondary cavities, which succeed them lower in the bone, is due. In the mean time the cartilage-cells have disappeared; and the bony cavities, as Mr. Tomes has pointed out, are filled with blastema, in which there are a few fibres and numerous granular corpuscles or cells resembling those seen in the intramembranous ossification; there are also many blood-vessels. In the end, some of the enlarged cavities remain to form the cancellated structure; while others, getting more and more filled up with concentric lamellæ, become Haversian canals; although the Haversian canals of the compact sides of the bone, it may be remarked, chiefly arise in another way, as will be afterwards described. In many of these cavities, the walls of the coalesced primary areolæ may long be distinguished, like little arches, forming by their union a sort of festooned outline, within which the new bony laminæ are situated." (Quain's Anatomy, p. clvii.)

The primary osseous deposit, as Mr. Tomes correctly remarked, is distinctly granular in its character, and is entirely destitute of lacunæ or canaliculi. The larger part of this, as we have just seen, is of temporary duration only, being removed before any subsequent deposit of osseous matter takes place; and it is difficult to recognise any of it in the fully-formed bone. The secondary deposit is quite transparent, and of a more uniform homogeneous aspect; and in this the lacunæ and canaliculi are first distinguishable. As to the mode in which these peculiar excavations originate, the observations of Messrs. Todd and Bowman, Mr. Tomes, and Dr. Sharpey, do not agree. The first-named observers speak of the lacunæ of the earliest secondary layers of bone as being evidently the nuclei of the cartilage-cells; the layers themselves being composed of cells which have been consolidated by ossific impregnation. The cancelli and canals then become filled with a granular blastema, like that out of which all the tissues are evolved; and from this new cells are generated, which undergo a similar change with the original cartilage-cells, their cavity being filled with osseous matter, whilst their granular nuclei remain as the lacunæ. All subsequent production of bone is supposed by these authors (Physiological Anatomy, p. 120,) to take place through the intermediation of "cartilage-cells," developed on the vascular surface, which become ossified in successive layers, their nuclei remaining to form the lacunæ, from the margin of which the canaliculi are elongated. We have been unable to find any ground for the opinion that the original cartilage-cells have any participation whatever in the production of the secondary osseous deposit with its lacunæ and canaliculi; as they seem to us to disappear altogether from the time when the primary osseous areolar structure begins to break down into the larger cavities, previously to its formation. Still less can we admit that any cells which may present themselves at a later period in the osteogenic process, have a title to be called cartilage-cells. Our authors here seem to have been warped by the prevailing idea that all ossification takes place in the substance of cartilage; so that wherever true bony structure exists, there must have been a preceding development of cartilage. Mr. Tomes now regards the lacunæ of bone as themselves being cells; and he considers that the bony matter which intervenes between them is produced by the simple consolidation of a granular deposit contained in the medullary cavities, which includes and unites them. (Cyclop. of Anat. and Physiol., vol. iii, p. 856.) The following is Dr. Sharpey's account of the process:

"Although certain appearances render it not improbable that there may be a layer of flattened and calcified cells next to the surface of the granular bone, I am nevertheless disposed to think that the subsequent and chief part of the deposit results from the calcification of successive layers of fibres, generated in the blastema and possibly derived from the granular cells, some cells being perhaps also involved along with the fibres, as in the ossification of the flat bones of the cranium; in short, it appears to me that the deposit in question is formed after the manner of the intramembranous ossification already described. I infer that such is the process, from the structure of the layers; for they are made up of fine reticulated fibres, like the lamellæ of perfect bone. On a careful inspection, and with a certain adjustment of the light, the little apertures of the canaliculi may be seen, and in many parts also fine striæ indicating the obliquely decussating fibres of the new-formed laminæ. The structure reminds us of the secondary deposit inside the oblong cells in the wood of coniferous trees, in which the ligneous matter is arranged in fibres, or rather in fine lines, running obliquely round the wall of the cell and crossing one another in alternate layers.

"The mode of production of the lacunæ, or so-called corpuscles of bone, is still an enigma in osteogeny, and I do not pretend to solve it. They are generally supposed to be derived from the cells of the soft tissue involved in the ossification by some sort of metamorphosis which has been variously conceived. Some suppose that the cell becomes the lacuna and sends out branches, like the pigment-cells, to form the canaliculi (Schwann). Others think that it is not the cell, but its nucleus, that undergoes this change, and that the substance of the nucleus is afterwards absorbed, leaving the lacuna (Todd and Bowman). Henle thinks that the lacuna is a cavity left in the centre of a cell which has been partially filled up by calcification, and that the canaliculi are branched passages, also left in consequence of the unequal deposition of the hard matter, as in the instance of the pore-cells of plants. As to this last opinion, it does not seem reconcileable with the structure of ordinary sound bone, and I am also led greatly to doubt whether the lacunæ and canaliculi are derived from cells or their nuclei in either of the two ways supposed. It has rather appeared to me as if they were little vacuities left in the tissue during the deposition of the reticular fibres, as open figures are left out in the weaving of some artificial fabrics (but not within a cell, as Henle imagined), and that thus the apposition of the minute apertures existing between the reticulations of the lamellæ gives rise to the canaliculi, in conformity with what has been already stated respecting their structure. At the same time it seems not unlikely that a cell or cell-nucleus may originally lie in the lacuna or central cavity, and may perhaps determine the place of its formation. Such is the view I feel disposed to take of the production of the lacunæ and canaliculi of ordinary bone, although I can by no means speak confidently on the point. In instances of what might be considered a more crude form of ossification, the mode is perhaps somewhat different. In the slow growth of bone, which encroaches on the attached surfaces of articular cartilages, the ossification would almost seem to be produced merely by the impregnation of the cartilaginous matrix with earthy matter (corresponding with the first step of the ordinary process,) and in this case the cells and clusters of cells being surrounded by the calcified matrix, may remain as little vacuities or lacunæ in the bone; but this, as well as the formation of lacunæ in the crusta petrosa of the teeth and the production of adventitious bony deposits in different textures, requires further investigation." (Quain's Anatomy, p. clix.)

With regard to the chief proposition here enunciated by Dr. Sharpey—that the osseous laminæ concentrically deposited within the Haversian canals have a fibrous tissue for their basis, and that this form of ossification is therefore essentially the same with that in which no cartilage has previously existed,—we are in full accordance with him. Whether the lacunæ are actual *cells* enveloped by the fibrous texture, or whether they are the spaces occupied by granular nuclei which do not undergo development into cells, is not perhaps a matter of much consequence; our own opinion leans to the latter view. But we cannot, with Dr. Sharpey, regard the canaliculi in the light of passages which *happen* to be left in the superposed laminæ of ossified fibres. Their whole arrangement is so regular, their inosculation with those of adjacent lamellæ is so remarkable, and their general distribution so characteristic, that we feel sure that their presence must be in some way determined by that of the lacuna; and that, whether this last be cell or nucleus, it sends out radiating prolongations whilst the surrounding substance is yet soft, which prolongations remain as tubuli when that substance has undergone calcification. This view is perfectly consistent with Dr. Sharpey's observations already quoted, with respect to the relation of the perforations to the fibres of the animal basis; for if the fibrillation of these take place *after* the bone-cells or nuclei have sent out their prolongations, a thin lamella peeled off in a direction transverse to the passage of the canaliculi will naturally show these as minute apertures in the meshes of the fibrous network, the fibres passing around them and inclosing them in their reticulations as described by Dr. Sharpey.

The most important of all the doctrines newly advanced by Dr. Sharpey, and one as to which we can most completely confirm the observations on which it rests, is this,—that the increase in girth of a long bone, like the increase in thickness in a flat bone, takes place by the progressive calcification of the inner portion of the periosteum, and that no generation of cartilage precedes the development of new osseous tissue in this situation. It is obvious that if this be true, as our own observations assure us that it is, independently of the authority of so cautious and trustworthy a physiologist as Dr. Sharpey, the whole controversy regarding the power of the periosteum to produce bone is at an end. The shaft of a long bone, the femur for example, is increased many times in diameter, from the period when it is at first moulded in cartilage, to the full development of the adult bone; and during its growth, the whole of the first-formed bone undergoes removal, as we shall hereafter see, to make way for the central medullary cavity of the enlarged shaft. Consequently the whole of the hollow cylinder or dense shaft of the adult bone must have been at first formed in the periosteum, though it no doubt undergoes subsequent changes by the progressive filling-up of the Haversian canals. It cannot be supposed, then, that a vascular membrane which is the immediate instrument of the regular production of osseous texture, should be anything else than the most important agent in its regeneration. The following is Dr. Sharpey's description of the process:

"It has been sometimes supposed that a formation of cartilage precedes the bone also in this situation; but such is not the case, for the vascular soft tissue in immediate contact with the surface of the growing bone is not cartilage but consists of fibres and granular corpuscles; in fact, the increase takes place by intra-membranous ossification, and accordingly the Haversian canals of the shaft are formed in the same way as those of the tabular bones of the skull,—that is, the osseous matter is not only laid on in strata parallel to the surface, but is deposited around processes of the vascular membranous tissue which extend from the surface obliquely into the substance of the shaft; and the canals in which these vascular processes lie, becoming narrowed by the deposition of concentric osseous laminæ, eventually remain as the Haversian canals.

"That the ossification at the periosteal surface of the bone does not take place in cartilage, may also be made apparent in the following manner. Strip off the periosteum from the bone at the end of the shaft and from the adjoining cartilage also, taking care not to pull the latter away from the bone. A thin membranous layer will still remain, passing from the bone to the surface of the cartilage; now, take a thin slice from the surface, including this membrane with a very thin portion of the bone and of the cartilage, and examine it with the microscope, scraping off the cartilage from the inside if it be too thick. You will then see that the superficial part or shell of the bone, if it may be so called, is prolonged a little way over the surface of the cartilage by means of pellucid coarsely-reticulated fibres of soft tissue into which the earthy deposit is advancing. These fibres are intermixed with granular corpuscles or cells, but form no part of the cartilage, and they are no doubt of the same nature as those seen in the intra-membranous ossification of the skull. Their reticulations are in most cases directed transversely, and sometimes they are little, if at all, in advance of the limit between the bone and cartilage. I have observed the structure here described in several bones of the (well advanced) fœtal sheep, also in the human scapula, humerus, femur, tibia and fibula, metacarpus and metatarsus, and it probably occurs in all the long bones." (Quain's Anatomy, p. 160.)

We have ourselves, in prosecuting our observations on the plan here suggested by Dr. Sharpey, been able in more than one instance to trace distinctly the continuity between the soft fibres of the internal periosteum, and the calcified fibres of the newly-forming bone; and we can now fully understand the rationale of the statement of Professor Goodsir, that it is impossible to separate the periosteum without removing along with it minute longitudinal, filamentary, or riband-shaped portions of the surface of the bone.

We thus perceive that microscopic research leads unequivocally to the following conclusions:—1. That true osseous tissue, with its characteristic lacunæ and canaliculi, may be formed from a membranous basis consisting of gelatinous fibres and interspersed cells. 2. That the osseous substance formed in cartilage is of an inferior character, being composed of an aggregation of granules without regular lacunæ or canaliculi; and that this is essentially the same, whether generated in the intercellular matrix of the temporary cartilage of the fœtus, where it constitutes the primary layers of bone, or in the substance of permanent cartilage during that process of abnormal consolidation which often takes place at a later period of life. 3. That the more perfect form of osseous tissue is always developed from a basis of fibres and interspersed cells, whether it is generated as a concentric deposit within the Haversian canals, or whether it is formed on the external surface of the bone. The development of new osseous tissue would seem to be ordinarily confined to these two situations.—There is no adequate reason to believe that bone enlarges by a process that can be properly called *interstitial* growth; or in other words, that the entire shaft swells by the deposit of new matter in every part of its substance. All our knowledge of its manner of increase would lead to the belief that its diameter is augmented by superficial deposit alone; its solidity being increased at a subsequent time by the narrowing of the Haversian canals, which is the result of the new osseous deposits concentrically made in their interior. The membrane lining the central medullary canal and cancelli, obviously belongs to the same category with the membranous lining of the Haversian canals and the periosteum, and is in fact continuous with these at many points; but it appears to be ordinarily concerned, rather in the absorption of bone than in its deposition, since we commonly find no new bone generated in the interior of the shaft or of the cancellated structure, but in its stead a positive removal of bony matter, in order that the enlargement of the cavities may keep pace with the general enlargement of the bone. We cannot help seeing, however, that the inner layer of periosteum, the lining membrane of the Haversian canals and cancelli, and the medullary membrane, all form one system, on which the whole development of the osseous substance depends, from the time when it was first marked out by the lamellæ deposited in the cartilaginous matrix. When we speak of membrane, we would be understood to include the peculiar cells, which are found between the membrane and the bony substance itself in all these situations, so long as the growth of the bone continues. It is only in bones which have nearly or quite arrived at their full growth, that we find the medullary cavity, the cancelli, and the Haversian canals, filled with fat-cells instead of with formative blastema; and it is then, too, that a layer of fat is commonly deposited on the external surface of the bone beneath the periosteum. What special purpose is served by this deposit, does not appear evident. There is no reason whatever to believe that it has any relation with the nutrition of the bone, since we find the bones of most birds destitute of it. We can only attribute its occurrence to the general tendency which exists in the adult to the deposition of fat in situations where it may be stored up out of the way, and especially in receptacles which have been employed during the earlier age of the animal for some purpose more immediately connected with its nutrition and development.

We shall now pass from this investigation to a review of the 'Théorie Expérimentale' which has recently been put forwards as a great novelty by M. Flourens. We need not say that anything which comes from a physiologist so distinguished is worthy of respectful attention; and we have accordingly perused his treatise (chiefly consisting of Memoirs presented at various times to the Academy of Sciences, of which M. Flourens is perpetual secretary) with every desire to profit by it. The experiments whose results it contains, have been very numerous and ingeniously varied; most, if not all of them, must have been attended with a great amount of suffering to the unfortunate subjects of them; and yet we cannot find that they contribute to establish a single general fact or doctrine which had not been distinctly enunciated by his predecessors. And yet with the self-complacent egotism so common among his countrymen, but with which we should have hoped to find so distinguished a *savant* somewhat less imbued, M. Flourens refers in almost every page to "ma théorie" as if it were distinct from every one that had been previously broached, and were destined to supersede them all,—instead of being, in fact, the doctrine of John Hunter, without those extensions and modifications which a more intimate knowledge of the processes concerned in the development and growth of bone have enabled the physiologists of this country to introduce into it. M. Flourens seems to have made up his mind to pursue the inquiry in a single method only,—the experimental one. For him microscopists have laboured in vain. He scorns to avail himself of their results. Instead of following out the natural processes, whose course, when attentively studied, reveals to us almost everything we can reasonably expect to know, he puts Nature (literally as well as metaphorically) to the torture, and seeks to learn from her efforts and struggles that which it only needed care and patience to ascertain from her calm and regular operations. French physiologists have always appeared to us to have an essentially wrong idea of the proper purpose of experiment. They forget the difference between the laboratory of the chemical or physical philosopher, and the living organized body. In the former nothing takes place of itself; whatever we desire to see must be arranged for; every process must be *set* in operation, whether it be a simple act of precipitation or evaporation, or whether it be a delicate and complex analysis, the results of which may be of the highest importance. But in the living body we have no need of this expenditure of labour in devising experiments, for every change that takes place under our scrutiny is in fact an experiment which Nature *has prepared* for us; and it is only after we have exhausted all the sources of information with which we are thus furnished, and find some points left in doubt on which a certain variation in the conditions of the phenomenon will probably enable us to satisfy ourselves, that we are justified (as it seems to us) either philosophically or morally in having recourse to this method of investigation. Our Gallic neighbours seem to have a natural turn for much that is repulsive to the feelings of an Englishman. They can starve multitudes of unfortunate animals to death with the same coolness that we should exercise in feeding them. They can lacerate the flesh, break or bore the bones, and dissect out the nerves, of a living animal, with as much nonchalance as we should feel towards a dead one. And they feel no compunction in repeating on any number of occasions, for the mere sake of display, experiments of such cruelty that we should have recourse to them only for the sake of acquiring information on some point of great importance which could not be otherwise settled, and should shrink from repeating them when no further instruction seemed likely to be derived from them. Perhaps this peculiar taste has had its advantages, in bringing to light a few facts which might not have been otherwise sought; but the discoveries to which it has contributed have been marvellously few, when compared with those which have resulted from what we deem a more philosophical method of inquiry into the secrets of Nature. The former researches of M. Flourens on the Functions of the Nervous Centres, are perhaps among the most valuable of all the contributions which the French experimental school has made to physiology; and yet we are much mistaken if the value of these be not hereafter estimated rather by their coincidence with the inductions founded upon extended anatomical inquiry, than by the apparent positiveness of their own indications.

In order that the position of the questions which M. Flourens has set himself to determine may be clearly understood, it will be necessary to revert for a brief space to the history of opinion regarding the growth and development of bone. The most ancient idea seems to have been, that the bones increased by *extension* in every direction, through the assimilation of nutritious fluid derived from the blood. It is not surprising that Clopton Havers should have sanctioned this view, when he traced the blood-vessels into the canals which bear his name, and thus showed how nourishment is supplied to every part of the bone. Duhamel was apparently led to investigate the subject, from an expectation that he should find something in the growth of bones analogous to the growth of trees by the addition of successive layers to their surface; and he was the first to propound the idea that the laminated structure of bone results from this mode of increase by *superposition*, the new layers being formed by the periosteum. Yet he does not seem to have entirely given up the notion that bone grows also by extension; this extension being produced by the *interposition* of new particles. At that time the idea of the possibility of the removal of bone already formed by the process of absorption, had not been entertained by physiologists; and there seemed no other way of accounting for the enlargement of the medullary canal, but by the notion that the entire cylinder expands by interstitial growth. Duhamel strongly upheld the instrumentality of the periosteum, as the agent concerned in forming the laminæ successively added to the exterior of the bone ; and on this and other points he was opposed by no less an authority than Haller, who maintained that the first formation of bone, whether in fœtal growth or in the production of callus, resulted from the consolidation of a gelatinous fluid, which first assumed the characters of cartilage, and then acquired those of bone by the ossifying process. Matters remained in this state when John Hunter took up the inquiry; and although the experiments which he seems to have made were few, and these imperfectly recorded, yet he introduced a new *idea* into the explanation of the process, which seems at once to have led to a considerable modification in the views then current on the subject, and which has left its impress on all subsequent opinion, in this country at least. This idea was the *removal by absorption* of osseous matter already deposited; so that whilst new matter was being added on the exterior, the form and proportions of the bone should remain the same. We unfortunately do not possess Hunter's own record of the experiments in question, or of his deductions from them. All our information on the subject is derived from a short paper drawn up by Sir Everard Home, which professes to give a summary of them. We need not say how much more satisfactory it would have been to have had Hunter's views expressed in his own terse and vigorous language. They are thus summed up by his successor:

"From these experiments he ascertained the changes which take place in bones during their growth and the readiness with which the materials of bone are absorbed; and from these facts laid it down as an established principle, that the absorbents are the agents by means of which the bones, during their growth, are modelled as it were and kept of the same shape. Bones, according to Mr. Hunter's doctrine, grow by two processes going on at the same time, and assisting each other; the arteries bring the supplies to the bone for its increase; the absorbents at the same time are employed in removing portions of the old bone, so as to give to the new the proper form. By these means the bone becomes larger, without having any material change produced in its external shape." (Hunter's Works, vol. iii, p. 318.)

Now although it is not here stated, in so many words, that the shaft of a bone grows in diameter by superposed layers, and that the medullary canal is enlarged by absorption, without any extension of the substance of the bone already formed, yet it is perfectly obvious that this would be Hunter's notion of the process in the particular case in question; it has been taught as Hunter's by British surgeons and physiologists from that time to the present; and it is clearly expressed by Professor Owen, who, in a note to the preceding passage, contrasts the Hunterian doctrine with that of Duhamel with express reference to the celebrated experiment of the latter, to which we shall presently make more particular reference.

"The difference between the Hunterian theory of the growth of bone, and that of Duhamel, will be readily appreciated by attending to the explanation given by Duhamel of the phenomena which he observed during his investigations on this subject. Let us, for example, select from the number of his ingenious and instructive experiments that in which he placed a ring of silver wire round the middle of the shaft of the thigh-bone of a young pigeon; and found at a subsequent period the ring in the medullary cavity of the bone, instead of embracing the exterior of the shaft where he had placed it. It need scarcely be observed that the Hunterian physiologist would explain these facts by stating that the arteries of the periosteum had deposited new bone on the external surface of the ring, while the absorbents had removed the old bone in contact with the internal surface of the ring, by which its relations to the osseous parietes of the femur became reversed. But this physiological view of the phenomena, arising out of a knowledge of the powers and actions of great and important vascular systems in the frame, was wholly unsuspected by and unknown to the predecessors of Hunter. Duhamel explains the facts on mechanical principles; assuming that the bony layers of the shaft of the thigh-bone were expanded by the interposition of additional osseous matter, and that the layers were cut through in this process of expansion by the unyielding wire which he had placed around them." (Hunter's Works, vol. iv, p. 318.)

These remarks, published as much as ten years since, sufficiently indicate the sense in which the imperfectly-expressed views of Hunter have been understood by British surgeons and physiologists; and it would be easy, if it were necessary, to adduce a large amount of testimony to the same effect from other quarters. Whether the lymphatic division of the absorbent system is as largely concerned in the removal as Hunter and his disciples have supposed, or whether it is not rather by the veins that the osseous substance of the interior of the bone is carried off, is a question of secondary importance. The main point is, that Hunter distinctly enunciated the doctrine of the growth of bone by *superposition only*, in opposition to Duhamel, who conceived that, besides the superposition of new particles, there is an interstitial deposition by which the entire substance of the bone undergoes expansion.

We are utterly at a loss to understand, therefore, on what pretext M. Flourens claims the slightest trace of originality for this doctrine, and trumpets it forth thoughout his treatise as "ma théorie." We can only account for the circumstance by that extraordinary blindness to the doings of scientific men of other countries, which has gained for French *savans* so unenviable a notoriety. We have a "glaring instance" of this tendency in the fact that in a recent number of the 'Comptes Rendus' of the French Academy, the photographic process, which was published some years since by Mr. Fox Talbot under the name of the calotype, which has been subsequently most extensively used in this country, and of which specimens were submitted to the Academy two years since by the author through M. Biot, has been *republished as original;* the only variations from Mr. Talbot's account being in the most trifling improvements of detail, which had in fact been in common use in this country at least twelve months previously. That we do M. Flourens no injustice in imputing to him a most unfair neglect of Hunter, to say nothing of Duhamel, will we think be sufficiently obvious from the following passages, which form the commencement of his treatise.

"This work is composed of four parts.

"The first is the exposition, and, I venture to say, the demonstration, of my theory on the formation of bone.

"The second is the reproduction, much abridged, of my experiments already published on the coloration of bones by madder.

"The third is the discussion of the theories which have preceded mine.

"The fourth is the examination of the important and novel problem of the relation of force with matter in living bodies.

"This problem, now for the first time truly set forth in science, will doubtless call the attention of physiologists to my experiments and my views." (pp. 17-8.)

The preceding sentences constitute the advertisement; the first chapter of the work itself, entitled "My Theory and the Experiments which demonstrate it," commences as follows:

"My theory of the formation of bone rests on the six following propositions:

"1st. That bone is formed in the periosteum.

"2d. That a bone increases in thickness by superposed layers.

"3d. That it increases in length by juxtaposed layers.

"4th. That the medullary canal is enlarged by the absorption of the internal layers of the bone.

"5th. That the heads of bones are successively formed and removed by absorption, to be again re-formed in accordance with the growth of the bone.

"6th. That the *continual mutation* of matter is the great and wonderful spring of the development of bone." (p. 2.)

In regard to the first proposition, M. Flourens generously admits that he was anticipated by Duhamel; for that sagacious physiologist expresses himself on the subject in a way that cannot be misunderstood, even going so far as to term cartilage a thickened periosteum. M. Flourens further accords to Duhamel the merit of exactness in his experiments: "I have," he says, "repeated them all; and everything which he says that he has seen, I also have seen." But, he asks, why is it that Duhamel's opinion has not been generally admitted; or rather, to speak more correctly, why is it that, commencing with Haller, it has been combated by almost every physiologist? The reason, according to M. Flourens, is to be found in the fact, that in judging of the opinions of Duhamel, no care was taken to repeat his experiments; and that these experiments, although exact as far as they went, were not sufficient to establish his views. Now upon this we have to remark that, although Haller and some subsequent physiologists may have found it difficult to reconcile Duhamel's views with their own preformed opinions, yet all who have themselves appealed to Nature have arrived at the same conclusion with Duhamel: so that, as physiologists have shown an increasing tendency to give up hypotheses in favour of facts, and to adopt actualities instead of abstract notions, the truth of Duhamel's doctrine on this point has been more and more fully recognised, in this country at least; and there has been of late so general a conformity in the doctrine that bone can be formed through the instrumentality of the periosteum, that the result of Dr. Sharpey's microscopic inquiries has been felt to be rather an anticipated consequence of Duhamel's doctrine, than a supplemental proof required for the corroboration of the latter. Still we are far from asserting that M. Flourens has not obtained by his experiments additional proof of a position that was still disputable. Our complaint is of the degree of credit he has taken to himself for having done so. And as any testimony derived from experiments on this subject must necessarily be very much of one kind, we do not think *his* confirmation of Duhamel's doctrine nearly so well adapted to convince those who may remain sceptical, as the observation of the actual process of the conversion of the periosteum into bone, carried on after the method of Dr. Sharpey.

In order to put in the clearest light the "great fact" of the formation of bone in the periosteum, M. Flourens did not confine himself, like Duhamel, to the simple experiment of fracturing the first bone he came to—the tibia for example—and then watching the results. In all such cases, the two ends of the bone are drawn together, or are even caused to override each other, by muscular contraction; and an effusion ensues, in the midst of which it is impossible to see clearly what subsequently takes place. "I say," observes our author, "to see clearly; for what is not seen clearly is not seen at all; and a great part of the art of experimenting consists in rendering the facts clear and precise." For the sake of avoiding the source of fallacy just alluded to, M. Flourens chose the ribs as the chief subjects of his experiments; since, their extremities being fixed, the divided ends are prevented from approximating when a portion of the bone has been removed. In these cases the periosteum that surrounded the portion of bone withdrawn, was left undisturbed; and the formation of new bone took place distinctly through its medium. It was found that the membrane first became greatly tumefied; that it then presented a cartilaginous structure; and that ossific deposits then began from centres or nuclei at points intermediate between the ends of the bone, gradually extending themselves so as to fill up the vacuity, and thus at last coming into contact with the original bone, at the cut extremities of which, according to M. Flourens, no new deposit is formed, so that it does not itself in any way participate in the reparative process. On this last point, however, other observers are at issue with him. In another series of experiments, M. Flourens aimed to give origin to a new bone, after the following extraordinary fashion: he drilled a hole in a bone, the tibia for example, and introduced into this hole a silver canula; he then partially detached a portion of periosteum from the surface, and carried it down this canula, so that it might come into contact with the medullary membrane of the interior of the bone; and the result was that this periosteum became thickened and cartilaginous, and then produced a new bone within the tube of the canula. Thus he considers that he has distinctly proved the fact of the power of the periosteum to produce bone. But we wonder that he should have been satisfied with this evidence, when the microscope, as Mr. Goodsir has shown, clearly reveals the impossibility of detaching the periosteum from a bone without carrying shreds of osseous tissue along with it; so that those who maintain that the periosteum alone cannot produce bone have a right to challenge M. Flourens to the proof that the periosteum alone was the agent in these cases.

The *second* proposition, that bone increases in thickness by superposed layers, is now so universally admitted that there appears to us still less ground for M. Flourens' enunciation of it as part of *his* theory; nor can we find that he has adduced a single new fact in its support. The experiment of placing a ring upon a growing bone, and watching its gradual inclosure by the newly-formed layers, until it is at last found within the medullary cavity, was first devised by Duhamel; and it has been so often repeated with the same result, and that result corresponds so precisely with other indications of the same truth, that M. Flourens might very well, we think, have spared himself the trouble, and the poor animals the pain, of its repetition.

The third proposition, that a bone increases in length, not by the growth of its entire substance, but by the formation of new osseous tissue near its extremities, is also, as our readers must be well aware, very far from possessing any claim to novelty; and the experiments upon which it is sustained by M. Flourens, are little more than a repetition of those of

Hales, Duhamel, and John Hunter. He seems, however, to have adduced additional evidence in support of the belief which had been formed upon other grounds, that the increase is chiefly effected by the progressive growth and ossification of the cartilage which continues to intervene between the shaft and the epiphyses of the bone, up to the period of its attaining its full development. This increase took place in M. Flourens' experiments, at both ends of the bone; but was rather greater at the upper than at the lower.

The enlargement of the medullary canal by the absorption of the inner layers of the bone, constitutes the *fourth* proposition. That the medullary canal increases in diameter with the shaft, so that in time it occupies a larger space than the entire solid bone originally did, is a matter of familiar observation; and it is proved, if proof were necessary, by the experiment of the ring, which, placed on the exterior of the very young bone, is found within the medullary cavity of the one more advanced in age. Duhamel seems to have thought that the hollow cylinder which forms the shaft, might expand by interstitial growth, and that the ring might cut through it (so to speak) the layers being successively divided by the ring from without inwards, and the section being then repaired; until all the layers had passed to the outside of it. But John Hunter in effect gave (as we have seen) the more philosophical explanation which is adopted by M. Flourens; namely, that the ring becomes imbedded by the formation of new layers upon the surface of the shaft, whilst the inner layers are removed by absorption; and M. Flourens has confirmed this view by a variation of the experiment, which was calculated to bring the question to an issue. Instead of employing a *ring* which presses and resists, and which might be imagined to cut through the dilating shaft, he introduced a thin lamina of platina beneath the periosteum, and found it (like the ring) covered by successive layers on its exterior, but brought nearer and nearer to the medullary cavity by the enlargement of the latter, until at last, from being applied to the outside of the shaft, it found its way to the interior of the cavity.

The *fifth* proposition enunciates that the heads of bones are successively formed and absorbed, to be again reformed with the progressive growth of the bone. M. Flourens does not tell us what he means by the term "têtes des os;" and if his statements apply to the epiphyses which are usually included under that designation, we apprehend that it will require modification. These epiphyses grow like the shaft of the bone; and as the great increase in length takes place between the epiphyses and the diaphyses, we apprehend that no greater amount of change need take place in them, than in the shaft itself. But from the manner in which M. Flourens states the case, it would almost seem as if he supposed that the heads of the bones had to undergo a continual absorption and renewal with the increase of the entire bone,—that which was once the head of the femur, for example, being changed in form so as to be incorporated with the shaft, and a new head being generated at its extremity. This must of course be the case with regard to processes which have no separate centres of ossification, such as the spine of the tibia, on which M. Flourens' experiments were made; but we apprehend that the epiphyses follow altogether a different rule, since they are for the time independent bones, and the increase of the shaft in length does *not* draw them into itself, but takes place at the expense of the intervening cartilage.

The *sixth* proposition is couched in the following grandiloquent terms. "The continual mutation of matter is the great and marvellous spring of the development of bones;" and this spring or source, we are presently told, was previously unknown. Possibly, it was a novel idea to M. Flourens; but we cannot see in what other light the experiments of Hales, Duhamel, Hunter, and their successors could be viewed, when interpreted by the light of modern physiology, which sees in this continual interchange only one example of what is constantly taking place throughout the body.

The second chapter is devoted to the truth of the "identity of the medullary membrane with the periosteum." M. Flourens first adverts to the experiments of Troja, who, having amputated a limb, destroyed the medullary membrane of the remaining bone, and then found that the old bone died and that a new bone was formed around it. An objection may be raised to the validity of any inferences drawn from such experiments, as to the functions of the medullary membrane; since the transverse division of the bone, and the removal of the lower part of the limb so much alters the condition of the remainder. Our author, therefore, adopted a different plan of operation; making a simple perforation in the bone, and destroying the medullary membrane of the entire cavity by means of a stylet introduced through the aperture. He found that an entirely new bone was then produced around the old one, which remained quite detached within it, undergoing gradual absorption from its external surface. He ascertained, further, that where the new bone had not begun to be developed, that which would afterwards become its medullary membrane was, in fact, the inner layer of the thickened periosteum. And he found strong reason to believe that the absorption of the old bone inclosed within this, was effected by the inner surface of the new medullary membrane; for this membrane presented a series of mamillary elevations and depressions, which correspond precisely with depressions and elevations on the surface of the bone, giving it an *eroded* aspect which seemed to be the result of the absorption of its material by the surface-action of the membrane. Not content with this method of proof, M. Flourens bored a hole in the tibia of a dog, and introduced into the medullary cavity of this bone one of the smaller ribs of a rabbit. He found that the medullary membrane became tumefied; and that the rib (as ascertained by numerous experiments of different duration) was gradually removed by absorption, until at last it disappeared almost entirely. Hence he concludes that the medullary membrane has a special power of *absorbing* bone. But he shows that this membrane has also the power of *forming* bone, by destroying the periosteum; in which case the shaft (or at least its outer layers) die, and new bone is formed in the medullary canal. We cannot refrain, however, from again expressing our astonishment that M. Flourens should have regarded either the experiment or the conclusion drawn from it as possessed of sufficient novelty to form part of *his* theory. He goes on to show that the removal of the old inclosing shaft, in this instance, is effected by a new periosteum, which grows over it without attaching itself; but, on the contrary, has the same kind of eroding action as the medullary membrane in the former case; and that, having eaten through the dead bone (so to speak) in various parts, it goes to attach itself to the surface of the new bone which this incloses, and, finally, becomes its proper periosteum. He further demonstrates the absorbing power of the periosteum, by introducing laminæ of an extraneous bone beneath the periosteum of a living bone; these gradually disappeared by the surface-action of the periosteum, just as in the former instance by that of the medullary membrane. From these and other considerations, it is concluded by M. Flourens, that the periosteum and medullary membrane are one and the same, the medullary membrane being in fact an internal periosteum;—a conclusion, be it observed, at which those who have been engaged in studying the minute structure of bone, and the mode of its development and continued nutrition, had arrived long since.

The Third Chapter relates to the formation of callus, which, according to M. Flourens, takes place in the periosteum, and nowhere but in the periosteum. The first of these propositions was sustained by Duhamel; and we do not find any new evidence in its favour in the present work. The second appears to us too exclusive; for although we are quite ready to admit the fallacy of the old notions that the callus is formed by the consolidation of effused fluid, or by a deposit from the extremities of the fractured bone, yet we cannot see any adequate reason for excluding the other membranous structures, which (on M. Flourens' own showing) form one and the same system with the periosteum, from a share in the process in cases in which they do not undergo the usual destruction or laceration. If the entire treatise had not shown a marvellous ignorance of the present state of physiological knowledge, we should have found a good subject for special animadversion in the following passage, wherein M. Flourens enunciates what he seems to regard as a great discovery:

> "Thus at last seen, for the first time perhaps, in its true light, the reunion of fractures by the formation of callus is no longer anything peculiar, exceptional, and mysterious in physiology.
>
> "Callus is bone and nothing but bone; and is bone which is formed where all bone is formed,—that is, in the periosteum.
>
> "The formation of callus is then but a particular instance of the general and ordinary process of the formation of bone." (p. 65.)

Although this statement is perhaps somewhat more formal and dogmatic than those to which we have been accustomed, yet it seems to us in no essential degree an advance upon the previous state of our knowledge; since all inquiry has tended to show that the reparative processes (wherever they are complete) take place on the same plan as the first development of the tissues which exhibit them: and the evidence of this has been peculiarly satisfactory in regard to bone, and does not seem to us in any way strengthened by the coarse experiments and observations of M. Flourens.

The Fourth Chapter consists of nothing else than a repetition of the statement, in a different form, of the fact that the periosteum has the power of reproducing bone, that it may regenerate any part or even the whole of a bone, and that if it be destroyed it is first regenerated and then reproduces bone. Our author shows in this and several other parts of his work a marvellous gift at *reproducing himself.* The following is his concluding summary:

> "The periosteum is then the material, the organ, the *stuff,* which serves for all these wonderful reproductions.
>
> "The periosteum is the organ which produces bones and reproduces them; and there is no other part of the animal economy which enjoys in so high a degree the faculty of reproducing itself.

"Some days are sufficient for its regeneration; and this regeneration is indispensable.

"A portion of the periosteum may be removed, and it reproduces itself; still more may be removed, and still more is regenerated; and so on.

"And now, after having brought into full view, after having demonstrated by so many different experiments, the surprising faculty, previously so little known (!) which bones possess of reproducing themselves, may I not be allowed to hope that this marvellous power will soon be a new agent in the hands of surgery? Without doubt. For I address myself to surgeons who observe, who think, and who do not see in surgery a mere routine method, but a science, a noble science; and who, even above that science, see humanity." (p. 72.)

We cannot depart from the strict verbal rendering of this grandiloquent passage without paraphrasing it altogether. We are totally at a loss to discover what are the new facts by which M. Flourens considers that he has given new powers to the surgeon. The ordinary course of disease has long since furnished examples in plenty of every principle which he advocates; and we do not see what can go beyond the reparative power which regenerates an entire bone, when the original one has been destroyed by necrosis,—a fact with which every surgeon is familiar, if not within his own experience, at any rate as an occurrence sufficiently common to find a place in every system of surgery.

The second part of the work contains the results of the madder-experiments performed by M. Flourens; and here, as elsewhere, we find marvellously little notice taken of the labours of his predecessors in the same line of inquiry; Belchier, the first discoverer of the peculiar affinity of this colouring-matter for the osseous texture, and Duhamel being the only authors referred to. Although Duhamel at first misinterpreted the results of these experiments, believing that the colourless rings of bone found after alternations of the madder-regimen with ordinary diet, were produced by the absorption of the colouring matter, he afterwards saw them in their true light, and perceived that the colourless rings were those which were formed during the intermission of the madder-regimen. We cannot see, therefore, on what ground M. Flourens thinks that by adopting this view (which was confirmed by Hunter and others) he has in any way contributed to the advance of science. Even the power of distinguishing by the use of madder not merely the additions made during a given time to the shaft of a bone, but also those made to its extremities, which is claimed by M. Flourens as a new observation, was distinctly indicated by John Hunter. He seems rather perplexed by the fact that the layer in immediate contact with the medullary cavity is often reddened by the same regimen as that which colours a layer that is being formed beneath the periosteum; and he also notices, without attempting to explain the circumstance, that the so-called colourless layers are seen with the microscope to exhibit traces of the reddening influence. These facts are easily understood, if the mode in which the osseous texture receives its nourishment be properly kept in mind. The vessels of bone are distributed on its external surface, on its internal surface, and through its small medullary canals and cancelli. In a very young bone, owing to the smallness of the shaft, and the openness of the Haversian canals, none of the osseous texture is far removed from the vascular surface; and the entire substance receives the maddertinge, within a day or even a few hours of the ingestion of that substance with the food. In the adult bone, on the contrary, it is difficult to produce any decided effect with madder. The thickness of the shaft withdraws the chief part of it from direct communication either with the periosteum or the medullary membrane; and the Haversian canals are so blocked up by the formation of concentric layers of bone, that the amount of vascular surface they afford is very small. The coloration of adult bones by madder is therefore confined to the portions in immediate contiguity with the periosteum and medullary membrane, and to the innermost of the concentric rings surrounding the Haversian canals; and in this last position it cannot be detected without the aid of a microscope. Now when a growing but not very young animal is fed with madder for a time, the layer of previously-formed bone in immediate contact with the medullary membrane will receive a tinge from it, and this may be so deep as to form a definite circle round the medullary cavity. At the same time a series of isolated deposits of the same kind will take place round the Haversian canals of the bone already formed; but these, although discernible with the microscope, will not give to the transverse section a tint visible to the naked eye. It will be in the new bone alone, formed beneath the periosteum during the continuance of the madder-regimen, that the entire osseous texture will be permeated by the colouring-matter.

The historical summary of opinions, which M. Flourens professes to give in his third part is meagre in the extreme. It takes cognisance of little else than the opinions of Duhamel and the objections of Haller; and although it includes a notice of the *experiments* of John Hunter, it says not a word of the *idea*, first applied by him to the interpretation of those and similar experiments, that the enlargement of the medullary canal is due to the absorption of the first-deposited bone.

We do not find it necessary to dwell on the "Examination of the relations between force and matter in living bodies," with which the treatise concludes; since nothing can possibly be more paltry than the manner in which the Perpetual Secretary of the French Academy here endeavours to gain the credit of originality by a mere piece of clap-trap. Our readers will scarcely believe us when we tell them that the only *idea* in the chapter thus grandiloquently headed is the following;—that as there is a continual mutation of matter in the living body, whilst the vital forces are comparitively unchanged, the *law* which fixes the relation of forces and matter in the living body is on the one part the *permanence of forces* and on the other the *mutation of matter*. We must own ourselves unable to perceive the novelty of either of the assertions embraced in this statement; as well as to understand how their juxtaposition can constitute a *law*. But upon the logic of science, our neighbours have always seemed to us to have very imperfect notions. Will it be credited, however, that M. Flourens assumes to himself the merit of *proving* the mutation of the materials of the living body?—a doctrine, he says, which with Buffon and Cuvier was the fruit of abstract meditation only. Truly he must be a man capable of entertaining but one class of ideas at a time; and that idea for the present is—*bone*. "There is nothing like it for proving everything; and everybody who did anything in the matter before me, did it so imperfectly that I must do it all over again and make it my own." This is a notion which seems to have possessed the mind of M. Flourens for some years past; and unless he redeems his character by a series of researches prosecuted in a more philosophical and less egotistical spirit, we shall be obliged to believe that the Perpetual Secretaryship of the French Academy has put an extinguisher upon all that was valuable in one of the most eminent of the French school of physiologists.

---

